# The immune regulatory role of lymphangiogenesis in kidney disease

**DOI:** 10.1186/s12967-024-05859-4

**Published:** 2024-11-22

**Authors:** Xiangheng Lu, Kuai Ma, Junyi Ren, Haoyu Peng, Jia Wang, Xiaoxiao Wang, Moussa Ide Nasser, Chi Liu

**Affiliations:** 1 Department of Ophthalmology, West China Hospital, Sichuan University, Chengdu, Sichuan China; 2 Department of Nephrology, Osaka University Graduate School of Medicine, Osaka, Japan; 3 School of Medicine, University of Electronic Science and Technology of China, Chengdu, China; 4 General Practice Center, Sichuan Academy of Sciences & Sichuan Provincial People’s Hospital, University of Electronic Science and Technology, Chengdu, 610072 China; 5 Department of Organ Transplantation, School of Medicine, Sichuan Provincial People’s Hospital, University of Electronic Science and Technology of China, Chengdu, China; 6 Department of Cardiac Surgery, Guangdong Provincial People’s Hospital (Guangdong Academy of Medical Sciences), Guangdong Cardiovascular Institute, Southern Medical University, Guangzhou, 510100 Guangdong China; 7 Department of Nephrology and Institute of Nephrology, Sichuan Provincial People’s Hospital, Sichuan Clinical Research Centre for Kidney Diseases, Chengdu, China

**Keywords:** Kidney lymphangiogenesis, Kidney disease, Lymphatic endothelial cell, Immune regulation, Immune cell trafficking

## Abstract

The renal lymphatic system is critical for maintaining kidney homeostasis and regulating the immune response inside the kidney. In various kidney pathological situations, the renal lymphatic network experiences lymphangiogenesis, which is defined as the creation of new lymphatic vessels. Kidney lymphangiogenesis controls immunological response inside the kidney by controlling lymphatic flow, immune cell trafficking, and immune cell regulation. Ongoing study reveals lymphangiogenesis’s different architecture and functions in numerous tissues and organs. New research suggests that lymphangiogenesis in kidney disorders may regulate the renal immune response in various ways. The flexibility of lymphatic endothelial cells (LECs) improves the kidney’s immunological regulatory function of lymphangiogenesis. Furthermore, current research has shown disparate findings regarding its impact on distinct renal diseases, resulting in contradictory outcomes even within the same kidney condition. The fundamental causes of the various effects of lymphangiogenesis on renal disorders remain unknown. In this thorough review, we explore the dual impacts of renal lymphangiogenesis on several kidney pathologies, with a particular emphasis on existing empirical data and new developments in understanding its immunological regulatory function in kidney disease. An improved understanding of the immunological regulatory function of lymphangiogenesis in kidney diseases might help design novel medicines targeting lymphatics to treat kidney pathologies.

## Introduction

The significance of lymphangiogenesis in various diseases has been extensively examined in recent literature. These studies have provided significant insights into the contrasting effects of lymphangiogenesis on disease pathophysiology. Lymphangiogenesis relies on the proliferation, migration, and differentiation of lymphatic endothelial cells (LECs). The cellular processes lead to the biosynthesis of lymphatic vessels, which transport excess fluid and regulate immune responses in the lymphatic system [1]. Recent studies utilizing genetic lineage tracing and single-cell RNA sequencing have demonstrated that stem/progenitor cells also play a crucial role in lymphangiogenesis [2]. Additionally, M1 macrophages have been shown to polarize and transdifferentiate into new LECs through activation of the vascular endothelial growth factor (VEGF-C)/vascular endothelial growth factor receptor 3 (VEGFR3) pathway [3]. Lymphatic vessel proliferation comprises healthy lymphangiogenesis (during wound healing and corpus luteum development) and pathological lymphangiogenesis. The latter is caused by pathological situations such as inflammation, tumors, and transplant rejection, among others [4–6]. Physiological and pathological lymphangiogenesis often entail the enlargement and sprouting of preexisting lymphatic vessels (LVs) rather than neolymphangiogenesis, which is more closely related to lymph node transfer [7]. The interaction between lymphangiogenesis and various clinical conditions has a complex effect on the organism. Advanced imaging and genetic approaches have made it possible to investigate specific structures and functions within the lymphatic systems in various diseases.

Lymphangiogenesis plays complex immune regulatory roles via various mechanisms, differing from the nuanced variations of microenvironments in tissues and organs. The newly formed lymphatic vessels can either enhance or inhibit the immune response [8, 9]. The lymphatic system maintains homeostasis and supports immune responses throughout various tissues and organs [10]. In both health and disease, the lymphatic system also plays a crucial role in regulating immune responses by directly influencing immune cells and coordinating their movement from tissues to draining lymph nodes (dLNs) [11]. The unique characteristics of lymphatic vessels in both health and disease demonstrate specificity related to tissue and organ types. The characteristics influence the varied outcomes of lymphangiogenesis in different disease contexts.

Within the kidney, lymphangiogenesis is closely linked to kidney tissue inflammation, fibrosis progression, and transplant rejection [12]. Evidence unveils that it can elicit dual-sided effects in various kidney pathologies [12, 13]. Studies have illuminated that kidney lymphangiogenesis exhibits an intricate immune regulatory mechanism capable of promoting or alleviating immune responses [14], depending on the specific kidney pathology under consideration. Emerging evidence suggests that, within kidney diseases, the distinct trafficking patterns of diverse immune cells and varying durations of different pathological conditions significantly contribute to the dual-sided effect of lymphangiogenesis [15, 16].

The kidney lymphatic system selectively transports renal interstitial fluid and immune cells. It actively contributes to the maintenance of kidney homeostasis and the orchestration of kidney immune response. Notably, preexisting lymphatic vessels within the kidney are predominantly distributed in the renal cortex and rare in the medulla. However, neo-synthesized lymphatic vessels can proliferate extensively throughout the kidney [17].

Lymphatic migrations of immune cells are regulated by Various chemokines, including chemokine (C–C motif) ligand 19 (CCL19), CCL21, and chemokine (C-X-C motif) ligand 12 (CXCL12). Additionally, several inflammatory and anti-inflammatory mediators, including Interleukin-1β (IL-1β), Tumor Necrosis Factor-α (TNF-α), Interleukin-10 (IL-10), and Transforming Growth Factor β (TGF-β), also involves in the regulation of lymphatic immune cell migrations [1].

To be specific, recent findings have uncovered that lymphatic vessels can suppress the expansion of CD8^+^ T cells [18, 19]. The interaction between Mac-1 on DCs and ICAM-1 on LECs mediates the adhesive interactions between DCs and LECs, thereby inhibiting the ability of DCs to induce T cell proliferation [20]. Moreover, chemokine receptor chemokine (C–C motif) receptor 7 (CCR7) expressed on DCs and its CCL21 produced by LEC are the main molecules involved in DC migration [21]. At the same time, reducing DCs is beneficial for slowing the progression of inflammation [22]. The reasons and mechanisms underlying this remarkable discrepancy in diverse kidney diseases require further investigation.

### LECs and their immune regulatory role

LECs are crucial in immune responses during inflammation, tumour, and other pathological conditions. Different subsets of LECs, including peripheral capillary LECs and lymph node LECs, have distinct functions. The primary functions of peripheral capillary LECs include fluid drainage, leucocyte transport, and participation in lipid metabolism. They also actively regulate the endocytosis of antigens, mediating by clathrin and caveolin. Interestingly, capillary LECs exhibit phenotypic adaptations in varying microenvironments [23]. Therefore, they can dynamically orchestrate the trafficking and activities of various immune cells. Among the intricate process of immune cell trafficking, lymphatic vessel endothelial receptor-1 (LYVE-1) makes the first adhesive contact between migrating immune cells and lymphatic endothelium, initiating the entry and trafficking of immune cells within afferent lymphatic vessels [24]. Moreover, capillary LECs secrete various chemokines to drive immune cell intravasation through a complicated process of actomyosin-mediated immune motility and β2 integrin activation during inflammatory status [24]. Among these cytokines, CCL21 is one of the most important and well-studied regulators. By binding to heparan sulfate within the extracellular matrix, CCL21 generates a hypotactic concentration gradient to promote the migration of diverse leucocytes, such as DCs, neutrophils, and monocytes, through interacting with CCR7 expressed on these immune cells [25–27]. Furthermore, accumulating data suggests numerous cytokines and chemokine/receptor combinations are involved in lymphatic migration. Immunosuppressive substances like IL-10 and TGF-β may prevent immune cells from migrating through the lymphatic system [28–30]. LN LECs exert varied functions after transporting molecules and cells to dLNs. These cells are pivotal contributors to immune surveillance in both health and disease. LN LECs and specifically distributed rapidly classify molecules [31–35]. These two types of LECs subtly regulate innate and adaptive immune responses [21, 22, 36–41].

### Cytokines and chemokines involved in lymphangiogenesis

Lymphangiogenesis is predominantly regulated by VEGF-C and VEGF-D, both of which directly bind with VEGFR-3 and the co-receptor neuropilin 2 (NRP2), expressed on the surface of LECs, subsequently inducing lymphangiogenesis [42]. Recent studies have revealed that several types of macrophages can promote lymphangiogenesis by secreting VEGF-C in various pathological conditions, including kidney damage and cardiac injury [43–47]. Cortical and medullary kidney tubules can secrete VEGF-C and VEGF-D within the kidney [48]. Studies suggest that VEGF-C has an essential role in the development of lymphangiogenesis, but its impact on the maintenance of lymphatic vessels might be limited [49]. Conversely, unlike VEGF-C, VEGF-D dominates the maintenance of lymphangiogenesis, which indicates a modulatory function of VEGF-D in its developmental stage [49]. Furthermore, TGF-β and connective tissue growth factor (CTGF) also contribute to the induction of lymphangiogenesis in kidney diseases, particularly in kidney inflammation and fibrosis [50]. Additionally, angiopoietins (Angs) are involved in the lymphangiogenesis mechanism. In fact, the Ang2/Tie/PI3K signaling pathway plays a crucial role in lymphangiogenesis; blocking this pathway leads to a decrease in VEGFR3 and inhibits lymphatic vessel formation [51]. Similarly, the transcription factors FOXC1 and FOXC2, which are part of the Forkhead box (FOX) family, positively regulate lymphangiogenesis. Studies have shown that FOXC1 and FOXC2 are essential for regulating the Ras/ERK signaling pathway during lymphangiogenesis, and the loss of FOXC1 and FOXC2 promotes excessive activation of ERK, leading to abnormal lymphangiogenesis [52].

### The immune regulatory functions of lymphangiogenesis in kidney diseases

#### Acute kidney injury

In many AKI animal models and AKI patient biopsies, VEGF-C and VEGF-D expression increased, as did robust lymphangiogenesis. Following kidney damage, inflammatory mediators such as Interferon-gamma (IFN-γ), TNF-α, and TGF-β promote lymphangiogenesis via several mechanisms [48, 53–56]. Functional neo-lymphatic vessels can manifest the same role as preexisting renal lymphatic vessels, promoting inflammation resolution through drainage of retained fluid, clearance of cellular debris, removal of pro-inflammatory cytokines and cells, and mobilizing immune cells [11, 48]. Macrophages are highly adaptable to transfer into various distinct phenotypes within the local microenvironment. Among these macrophages, M1 macrophages are predominantly infiltrated during the AKI process, primarily promoting inflammation response and inducing kidney injury [57]. They enhance the synthesis of new lymphatic vessels in renal inflammation and fibrosis microenvironment due to elevated expression levels of VEGF-C induced by TGF-β [49]. Recent evidence has demonstrated that M1 macrophages can directly contribute to the synthesis of new lymphatic vessels through transdifferentiating into LECs [3, 58]. Increased levels of VEGF-C directly suppress macrophage autophagy, which prompts M1 macrophage polarization into LECs [3].

Lymphangiogenesis adversely affects the AKI process, exacerbating renal inflammation [56, 59, 60]. In general, AKI-induced lymphangiogenesis can exert a dual-sided impact on the kidney. In AKI, kidney lymphangiogenesis also acts as an immunological regulator to balance immunity and immune pathology despite clearing excessive fluids, noxious stimuli, and inflammatory cells. Neo-synthesized lymphatic vessels can either induce or suppress the immune response in AKI models, regulating local and systematic immune systems through diverse mechanisms [61]. Firstly, it significantly enhances lymphatic flow, actively participating in the early immune regulation process after AKI. During AKI’s initiation, maintenance, and regression process, abundant immune cells, including inflammatory monocyte, neutrophil, lymphocyte and natural killer cells, orchestrate the overall immune response [62]. Therefore, lymphangiogenesis can mitigate kidney tissue inflammation by properly removing infiltrated immune cells at the injury site (Tables 1, 2).

**Table 1:** The role of important immune cells that involve in kidney disease

	Acute kidney injury	Kidney fibrosis	Hypertensive nephropathy	Diabetic kidney disease	IgA glomerulonephritis	Lupus nephritis	Polycystic kidney disease	Kidney transplantation (allograft rejection)
Immune cells								
Macrophage	Macrophages can induce kidney lymphangiogenesis through upregulated expression level of VEGF-C and can also directly promote lymphangiogenesis through transdifferentiating into LECs[43, 114, 116]							
	M1 macrophages enhance lymphangiogenesis and promote kidney inflammation response, aggravating kidney injury[51, 53]	M2 macrophages promote tissue fibrosis process in the context of chronic kidney disease and kidney fibrosis [53]	Macrophages aggravate kidney damage and elevating blood pressure level. Meanwhile, hypertensive condition activates TonEBP in VEGF-C-expressing macrophages, leading to expression of VEGF-C, which promotes lymphangiogenesis, subsequently enhancing removal of excessive interstitial fluid [102]	M1 macrophages are the predominant macrophages within DKD, which enhance the inflammation response through secreting pro-inflammatory cytokines and presenting antigens to initiate immune response, ultimately causing renal injury. M2 macrophages can suppress the immune response within DKD through expressing anti-inflammatory cytokines [155–157]	CD137L-secreted macrophages interact with CD137 on LECs, inducing lymphatics autophagy and lymphangiogenesis [140]	M2 macrophages promote tissue fibrosis process in the context of chronic kidney disease and kidney fibrosis [53]	M2 macrophages are predominant macrophage within PKD. They promote the growing of cysts [144]	
Cytotoxic T cell	Activated CD8 + T cells directly promote immune response at injury site. In contrast, infiltrated CD8 + T cells can induce LECs to produce PD-L1, further suppressing local CD8 + T cell [67]							
		IFN-γ-producing CD8 + T cells inhibit the differentiation of CD4 + T cell into Th2 cells, subsequently control kidney inflammation and fibrosis [88, 89] CD11c + CD8 + T cells induce fibroblast apoptosis in obstructed kidney disease [88, 89]					CD8^+^ T cells present a protective role in the context of PKD [146]	
Th cell	Th17 cells recruits neutrophils and other inflammatory cells to significantly aggravates kidney tissue damage and accelerates the progression to CKD [65, 66]	Th17 cells and Th2 cells present a profibrotic effect on injured site [86, 87]	Th1 cells and T17 cells secrete pro-inflammatory and pro-hypertensive cytokines, ultimately causing kidney tissue damage and sodium retention [110]	Th17 and Th1 cells conduct a strong pro-inflammatory response within DKD. Th 2 cells mainly suppress Th1 activation and therefore the inflammatory response, ameliorating the progression of kidney fibrosis [87, 158]	The distinct polarization patterns of Th1, Th2, and Th17 cells within IgAN lead to abnormalities of lymphocyte function. However, the differentiation of Th17 might improve the abnormal humoral immunity[137–139]			Th1/Th2 balance predominantly regulates transplant rejection [131]
Treg cell	Treg cells inhibit immune response by secreting suppressive cytokines and direct communication between cells. During AKI, T reg cells inhibit inflammation and facilitate tissue repair to reduce renal injury [72]	Treg cells protect the kidney against fibrosis progress [85]	Treg cells alleviate kidney inflammation response and improve sodium retention [72, 111]	Treg cells improve insulin resistance and suppress the immune response in kidney to ameliorate DN pathogenesis within the hyperglycemia setting [112]	Treg cells elicit attenuated immunosuppressive function within IgAN [159]			Treg cells mediate between transplant tolerance and rejection [160]
B cell		Recruitment of B cells in renal tissue exacerbates kidney fibrosis via increasing macrophage infiltration [82], inhibiting T cell differentiation and activation [83, 84]			Abnormally-functioned B cells exert a pathophysiological role within IgAN [133], [161]			B cells are crucial both in T cell-mediated graft rejection and antibody-mediated graft rejection [162]
Dendritic cell	CCR7 + dendritic cells at injury site dispose antigens and then present these antigens to CD8 + T cells in kidney draining lymph node, activating the activation and homing of antigen-specific CD8 + T cells. During AKI, dendritic cells contribute to a positive feedback that aggregates inflammation response [48, 57, 71, 72]

**Table 2:** Two-sided consequences of immune regulation through lymphangiogenesis in kidney diseases

	Beneficial effect	Detrimental effect	Refs
Acute kidney disease	Lymphangiogenesis enhances lymphatic flow and LEC-dependent regulation of lymphatic migration, promoting removal and migration of infiltrated immune cells to alleviate kidney inflammation	It enhances the positive feedback between CD8^+^T cell activation progress and inflammation by promoting dendritic cell migration It reduces the accumulation of Treg cells, which aggravates tissue injury PD-L1 produced by LECs inhibits activation and accumulation of local CD8^+^T cell	[59, 60, 62–70]
Kidney fibrosis	Lymphangiogenesis relieves intrarenal immune response and alleviates kidney fibrosis, primarily through clearance of TGF-β and macrophages. Increased removal of infiltrated γδ T cells, Th17 cells and CD4 + T cells through newly synthesized lymphatics can also contribute to this anti-inflammatory effect	The pro-inflammation positive feedback mentioned above dominantly regulates the immune response within kidney fibrosis Reduction of Treg cells, IFN-γ-producing CD8 + T cells and CD11c + CD8 + T cells also attributes to the fibrotic process	[47, 48, 86–89]
Hypertensive nephropathy	Lymphangiogenesis improves resolution of sodium retention and enhances clearance of infiltrated immune cells It also directly inhibits DCs activation by reducing Na + retention	It only exerts a limited trafficking of immune cells, which cannot overall alleviate kidney tissue damage, possibly due to the excessive accumulation of several immune cells within HNT It can reduce the accumulation of anti-inflammatory immune cells The pro-inflammation positive feedback mentioned above enhances the inflammatory response, accelerating the progression of HTN	[93, 95, 103, 105, 107–109, 112]
Diabetic kidney disease	Lymphangiogenesis transports inflammatory cells, clearing interstitial oedema, subsequently protecting the kidney allograft. [127–129]	Chronic hyperglycemia condition markedly leads to abnormally-structured lymphatic vessel, which ultimately aggregates kidney tissue damage The pro-inflammation positive feedback mentioned above leads to constant and intensive inflammation response, exacerbating the damage of kidney structure and function	[46, 52, 71, 114]
IgA glomerulonephritis		Dysfunctional lymphangiogenesis aggravates kidney inflammation and tissue injury, driving to the progress of kidney fibrosis	[5, 140]
Lupus nephritis		The positive feedback mentioned above probably exacerbates kidney inflammation and the progress to fibrosis	[142]
Polycystic kidney disease	Lymphangiogenesis transports accumulated fluid in cysts and inhibit cyst progression. It may also alleviate PKD through transportation of inflammatory cells and M2 macrophages surrounding the cysts		[144]
Kidney transplantation	Lymphangiogenesis transport inflammatory cells, clearing interstitial oedema, subsequently protecting the kidney allograft [127–129]	Increased accumulation of antigen presenting cells can aggregate graft rejection	[150–152], [158]

Despite lymphatic flow, some immune cells, including T cells, B cells, and DCs, can also directly regulate LECs-related signal pathways [63, 64], contributing to the inflammatory progress. Entry of naïve T cells to afferent lymphatics is regulated by the S1P (sphingosine-1-phosphate) receptor pathway [65], while memory T cells also possess CCR7, which binds with CCL21. Immune cells with CCR7 can also be regulated by the CCL21 gradient expressed by LECs, actively migrating to dLNs through afferent lymphatics [66]. Of note, current evidence has demonstrated that enhancing antigen-specific T helper cell 1 (Th1 cell) cell migration from tissues to dLNs accelerates the resolution of inflammation. In the setting of AKI, upgraded infiltration of Th cells (T helper cells), particularly T helper cell 17 (Th17 cell), has been observed [67]. Th17 cell, which aggravates tissue injury by recruiting neutrophils and other inflammatory cells, is the most abundant lymphocyte infiltrated at the injury site following AKI in mice [68, 69]. Additionally, intestinal flora-derived Th17 cells have been proved to migrate to the kidney in kidney disease. They enter peripheral blood circulation through lymphatic vessels regulated by the S1P-R1 pathway [16]. Subsequently, they return to the renal inflammation site through blood circulation, further exacerbating the inflammatory response [16]. Increased reduction of Th17 cells through kidney lymphangiogenesis may significantly mitigate kidney damage, alleviating AKI and the following progression to chronic kidney disease (CKD). However, further studies are required to demonstrate whether lymphangiogenesis can aggravate kidney damage by regulating these Th cells.

Moreover, due to the adaptiveness of LECs, lymphangiogenesis can also directly suppress the local CD8^+^ T cells during inflammation. This intricate mechanism has been well-studied in the setting of skin lymphangiogenesis. Lymphatic endothelial cells (LECs) largely express nonhematopoietic programmed death-ligand 1 (PD-L1) to limit local CD8^+^ T cell effectors to functioning in inflamed skin and melanoma [70]. Despite PD-L1-dependent inhibition of T cell antigen receptor (TCR) signaling, evidence supports that PD-L1 can regulate lymphocyte migration through endothelial and epithelial barrier tissues [70], which indicates that PD-L1 may directly regulate T cell transendothelial migration without antigen presentation mechanism. Similarly, the activated PD-1 signalling pathway in the kidney protects the ischemia–reperfusion-induced AKI mouse model [71]. At the beginning of inflammation, infiltrated antigen-specific CD8^+^ T cells produce IFN-γ, which directly induces PD-L1 expression in adjacent inflammation-induced lymphatic vessels. Abundant PD-L1 expressed by LECs limits the further accumulation of CD8^+^ T cells at the injury site, alleviating kidney inflammation.

**Fig. 1:**
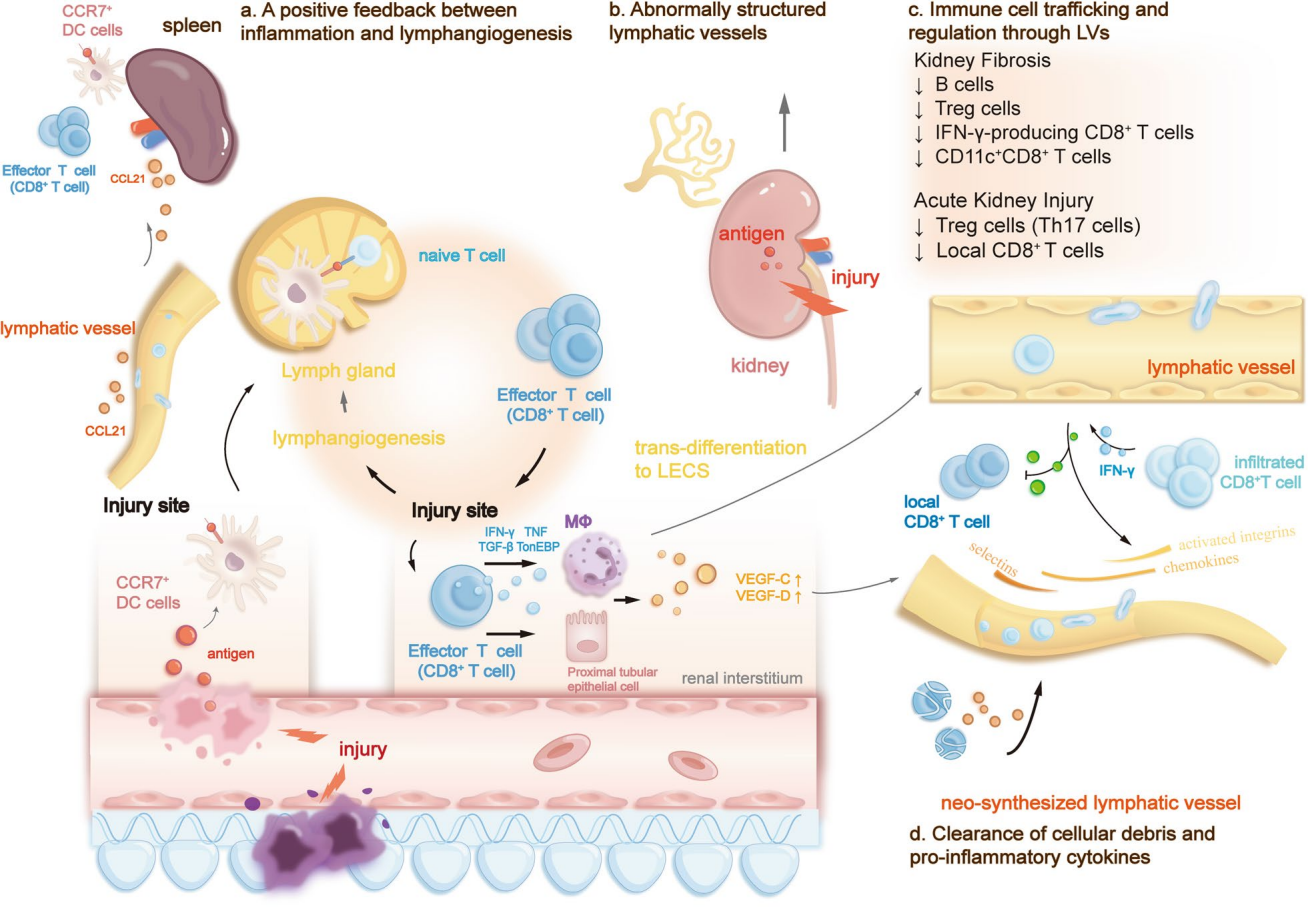
Immune regulation roles of lymphangiogenesis in inflammation settings. **a**. During kidney inflammation, lymphangiogenesis is significantly induced, and LECs overexpress chemokine CCL21, which promotes recruitment of CCR7^+^ immune cells to kidney dLNs through lymphatic vessels. Increased migration of CCR7^+^ dendritic cells with antigen presented promotes antigen-specific CD8^+^ T cell proliferation and homing to inflammation site. These infiltrated CD8^+^ T cells released inflammatory cytokines including interferon γ (IFN-γ), tumor necrosis factor-α (TNF), transforming growth factor β (TGF-β) and transcription factor of tonicity-responsive enhancer-binding protein (TonEBP). These cytokines promotes macrophages and proximal tubular epithelial cells to express several factors including VEGF-C and VEGF-D that eventually further prompt kidney lymphangiogenesis. **b**. Constant inflammation microenvironment results in abnormally-structured lymphangiogenesis, which aggravates inflammation response in kidney. **c**. Immune regulation role of lymphangiogenesis functions differently in multiple pathological conditions, resulting in diverse immune microenvironments. In kidney fibrosis, reductions of B cells, Treg cells, IFN-γ-producing CD8^+^ T cells and CD11c^+^CD8^+^ T cells are shown. And in acute kidney injury, accumulations of Treg cells (Th17 cells) and local CD8^+^ T cells are inhibited. Of note, despite actively regulating immune cell migrations, during inflammation, infiltrated CD8^+^ T cells released IFN-γ, inducing PD-L1 expression by LECs, further inhibiting local CD8^+^ T cell effector function, reducing accumulation of local CD8^+^ T cell and alleviating kidney damage and progression of kidney fibrosis. Lymphangiogenesis significantly aids to this mechanism through enhanced immune cell trafficking. **d**. Lymphangiogenesis promote clearance of cellular debris, pro-inflammatory cytokines. In AKI, it significantly reduce the level of TGF-β to suppress the inflammatory response in kidney

In the context of AKI, lymphatic migration of immune cells affects local immunity bidirectionally. Current evidence has demonstrated that lymphangiogenesis can also be detrimental to AKI [11, 72]. This detrimental impact closely correlates with positive immune feedback (Fig. 1) that enhances immune cells’ constant migration and activation at the injury site. During kidney inflammation, the increased level of CCL21 that is overexpressed by preexisting LECs, along with other chemokine and integrin pathways, promotes kidney dLNs and spleen to recruit more CCR7^+^ immune cells through afferent lymphatic vessels [73, 74]. The significantly elevated recruitment leads to systemic expansion of lymphocytes [56]. Within kidney dLNs, CCR7^+^ DCs present antigens of injury sites to CD8^+^ T cells, promoting T cell proliferation in dLNs. After which, the activated CD8^+^ T cells return to injury tissue via blood circulation, releasing inflammatory cytokines including IFN-γ, TNF, TGF-β, and TonEBP (transcription factor tonicity-responsive enhancer-binding protein), and therefore aggravating the inflammatory infiltration and injury in the kidney. Also, it released inflammatory cytokines, further prompting kidney lymphangiogenesis and lymphatic, immune cell migration. This positive feedback between kidney dLNs and the injury tissue regulates the immune response in AKI. Both disrupting this loop (whether by removal of renal dLNs or inhibiting DCs recruitment and inhibiting kidney lymphangiogenesis can facilitate the progression of kidney injury [56, 59]. Besides DCs migration, T cell migration also plays an essential role in AKI. As regulatory T cells (Treg cells) reduce renal injury by inhibiting inflammation and facilitating tissue repair during AKI, obstructing the migration of Treg cells to dLNs reduces inflammation [75]. Therefore, lymphangiogenesis, which greatly promotes Treg cell migration from the injury site, can enhance the inflammation response, subsequently exacerbating tissue injury in AKI. Despite T cells, lymphangiogenesis can promote B cells egressing to dLNs [1], which may also contribute to the integral immune regulation impact of lymphangiogenesis in AKI.

To date, the integral effect of kidney lymphangiogenesis on AKI remains controversial. Despite protective or detrimental impacts, the argument that kidney lymphangiogenesis only acts as a passive response to AKI also exists. In an adriamycin-induced mouse model, it was proved that inhibited lymphangiogenesis did not affect renal inflammation and fibrosis progression [76]. Dynamic immune regulation mechanisms reacting to the different microenvironments of diverse AKI models and the duration of AKI may explain the contradictory outcomes of kidney lymphangiogenesis in AKI. Although these immune regulation mechanisms are widely triggered in a large number of diseases, evidence has suggested that lymphangiogenesis triggered by different stimuli differs in its immune regulation impacts to some extent. For instance, B cells-driven dLNs lymphangiogenesis significantly contributes to immune cell migration [77], while tumor-induced dLNs lymphangiogenesis mainly enhances lymph flow and metastasis [78, 79]. Nonetheless, currently, few studies focus on the complex mechanisms and the balance between opposite outcomes beneath AKI-induced lymphangiogenesis. Though lymphangiogenesis is found in different AKI models, it manifests divergent impacts on different models, including ischemia–reperfusion injury-induced, unilateral ureteral obstruction-induced and several toxin-induced models. However, its specific mechanisms are still unknown [80]. Given the varied pathogeneses underlying these models, future investigations need to elucidate lymphangiogenesis's distinct roles in these AKI models.

Additionally, since UUO can directly cause great urinary retention, which might confound the factors that trigger the initiation and maintenance of lymphangiogenesis, it might not be the ideal AKI model to study these intricate mechanisms involved in kidney lymphangiogenesis [48].

Hemolytic uraemic syndrome (HUS) is a group of disorders including AKI, thrombocytopenia and microangiopathic hemolytic anemia [81]. It is a rare but often life-threatening syndrome that various infective and non-infective reasons can induce. Shiga toxin-associated HUS is the most common type [82]. In general, all the non-infective types refer to atypical HUS, which is often associated with dysregulation of the complement system [83]. The consistent features of all types of HUS manifested in the kidney include aberrant immune cell populations and remarkable renal inflammation [81, 82, 84]. To date, classical treatments are limited to supportive options, and few targeted therapies are implemented in the clinic except anti-complement therapy. A recent study reported that ibrutinib and acalabrutinib (Bruton’s tyrosine kinase inhibitors) significantly reduced immune cell invasion and ameliorated disease progression [82]. Bruton’s tyrosine kinase inhibitor is crucial for innate immune response by regulating the recruitment and function of immune cells [85]. Therapies focused on promoting the growth of lymphatic vessels may be a viable alternative approach due to the unique feature of highly increased immune cell recruitment in the kidney. In contrast to other AKI scenarios, the process of lymphangiogenesis may significantly enhance the movement of immune cells, which in turn worsens the inflammatory response in the kidney. However, more studies are necessary due to limited research, especially on lymphangiogenesis in HUS.

Further studies are urgently needed to provide a clearer acknowledge of how newly synthesized lymphatic vessels act as an immune switch, specifically in the setting of AKI, which can yield novel insights to alleviate AKI through utilizing protective aspect of kidney lymphangiogenesis or avoiding detrimental actions of kidney lymphangiogenesis.

#### General function of lymphangiogenesis in chronic kidney disease

DN, IgAN, and LN are significant contributors to the progression of chronic kidney disease (CKD) and subsequent renal fibrosis [36]. CKD is a progressive condition characterized by gradually losing kidney function over time. The key pathological features of CKD are renal fibrosis and inflammation, which involve the excessive accumulation of extracellular matrix proteins in the kidney, leading to scarring and structural damage [40]. In CKD patients, diverse types of immune cells are infiltrated, mostly including macrophages, T cells, DCs, and mast cells [86]. The protective function of lymphangiogenesis is primarily exhibited through the clearance of local inflammatory factors and immune cells in the kidney.

Lymphangiogenesis reduces macrophage infiltration at the injury site and decreases the level of TGF-β, subsequently relieving intrarenal immune response and retarding the fibrosis progression [87]. Unlike AKI, M2 macrophages are the predominant phenotype within kidney fibrosis, which promotes tissue fibrosis in chronic kidney disease and kidney fibrosis [57]. Additionally, TGF-β serves as a master regulator during the progressive process of CKD. Physiologically, TGF-β in the kidney is responsible for the maturation of immune cells and regulating immune tolerance and response. However, overexpression of TGF-β induced by kidney inflammation disrupts the immune balance and accelerates the progression of kidney fibrosis. Additionally, lymphatic retention and interstitial fluid accumulation also increase expression level of TGF-β [88]. Therefore, clearing excessive fluid through kidney lymphangiogenesis also reduces TGF-β, which subsequently activates immune cells, including macrophages, reducing the progression of kidney fibrosis.

Besides clearing overexpressed TGF-β and infiltrated macrophages, recent studies elucidate lymphangiogenesis’s important immune regulation role by removing B cells in the kidney to mitigate fibrosis. B cells can affect kidney fibrosis through cytokine production and interactions with macrophages, T cells, and fibroblasts. Accumulating evidence demonstrates that depletion of B cells exhibits a protective effect towards kidney fibrosis in animal models. B cell-deficient mice were resistant to UUO-induced renal interstitial fibrosis [89]. Infiltrating B cells in kidney lesions exacerbate fibrosis by secreting various chemokines, including CCL2 and chemokine CCL7. Evidence demonstrates that enhanced recruitment of B cells in renal tissue exacerbates CKD via increasing macrophage infiltration [89], inhibiting T cell differentiation and activation [90, 91]. In CKD progress, lymphangiogenesis promotes B cell's egress to dLNs, decreasing B cell accumulation in the kidney. However, whether lymphangiogenesis can protect the kidney directly through reducing renal B cell accumulation and profibrotic chemokines secreted by B cells remains to be established. At first, overexpression of CCL21 by newly formed lymphatic vessels enhances the recruitment of CCR7^+^ DCs in dLNs. It eventually systematically promotes antigen-specific CD8^+^ T cells infiltration and kidney inflammation response, further inducing lymphangiogenesis. This chronic and systematic immune response significantly aggravates the inflammation and fibrosis progression. Suppressing the recruitment of CCR^+^7 DCs alleviates the infiltration of inflammatory cells infiltration and the progression of kidney fibrosis [16].

Reducing local immune cells due to lymphangiogenesis can also be maleficent in kidney fibrosis, inducing CKD. Studies have demonstrated that Treg cells protect the kidney against fibrosis progress [92], while Tγδ cells, Th17 cells, and CD4^+^ T cells present a profibrotic effect on the injured site [93, 94]. Therefore, reducing these anti-fibrotic immune cells due to kidney lymphangiogenesis can accelerate the progression of kidney fibrosis. Contrary to conventional acknowledge, subsets of CD8^+^ T cells, including IFN-γ-producing CD8^+^ T cells and CD11c^+^CD8^+^ T cells, also exert an anti-fibrotic and renal protective role in the kidney fibrosis setting. IFN-γ-producing CD8^+^ T cells inhibit the differentiation of CD4^+^ T cells into Th2 cells, subsequently controlling kidney inflammation and fibrosis, while CD11c^+^CD8^+^ T cells induce fibroblast apoptosis in obstructed kidney disease. These CD8^+^ T cells reduce myofibroblasts accumulation, which is one of the principal pathologies of CKD [95, 96].

Besides, sustained inflammation in the kidney may eventually result in the abnormal structure of newly synthesized lymphatic vessels [97], which destructs lymphatic vessels' functions, aggregating the progression of kidney inflammation and fibrosis (Fig. 1).

#### Diabetic kidney disease

Chronic hyperglycemia can lead to progressive diabetic kidney disease (DKD), which is the leading cause of end-stage renal disease (ESKD) in many countries. Excessive lipid accumulation in kidney tissue stimulates the expression of TGF-β and TNF-α, consequently resulting in an inflammatory response, eventually leading to severe diabetic renal damage [98].

As for the reason of lymphangiogenesis in DKD patients, it occurs due to a hyperglycemia-induced pro-inflammatory environment [99, 100], which creates a positive feedback loop between kidney tissue and dLNs, leading to an intensified inflammatory response in the kidneys [56, 74]. Additionally, the markedly elevated expression of VEGF-C in hyperglycemic conditions [53], while excessive ROS production from lipotoxicity induces apoptotic cell death, damaging the lymphatic endothelium and further promoting abnormal lymphangiogenesis [101].

In the context of the immune mechanisms of lymphangiogenesis in DKD, DC cells and macrophages play an indispensable role. Under hyperglycemia, the amount of DCs greatly increases, and danger-associated molecule patterns (DAMPs) interact with pattern recognition receptors on kidney DCs, activating CD8^+^ T cells, a feedback loop between kidney lymphangiogenesis and immune response. The macrophage population increases heavily in the glomeruli and tubulointerstitial within human type 2 diabetes. The intrarenal macrophages that were recruited primarily underwent polarization towards the M1 subset. This polarization resulted in heightened expressions of both systemic and renal cytokines, such as MCP-1 and TNF-α. Consequently, neo-lymphatic vessels developed, leading to the ultimate progression of glomerulosclerosis and tubulointerstitial fibrosis. Studies have demonstrated that macrophage infiltration prevention alleviates DKD progression [102].

In DKD condition, the newly synthesized lymphatic vessels in the kidney are dilated, with characteristics of hypertonicity and aberrant functions [97]. Notably, abnormal lymphangiogenesis might also partly damage preexisting lymphatic vessels’ function [99].

#### IgA nephropathy

Current studies have suggested that VEGF can manifest protective or detriment effects in glomerulonephritis [103–105]. These studies mainly focused on the roles of VEGF-induced angiogenesis in diverse chronic glomerulonephritis models; nonetheless, only a few studies targeted lymphangiogenesis. A recent study has provided evidence of a correlation between kidney lymphangiogenesis and clinical outcomes of IgA [106]. The increased lymphatic vessel density significantly correlates with more serious renal function injury and earlier progression to ESKD [48, 106]. Previous studies proved that the density of lymphatic vessels manifested as a promising prognostic value to predict the risk of ESKD for IgAN patients [106]. Similar to IgAN, a recent study demonstrated that the increased kidney lymphatic vessel density correlated with poor outcomes in crescentic glomerulonephritis [107]. Research has suggested that increased immune cell infiltration in crescentic glomerulonephritis is highly associated with kidney lymphangiogenesis [107, 108]. However, whether increased infiltration of immune cells induced by lymphangiogenesis plays a crucial part in IgAN remains uninvestigated. Emerging evidence has unveiled attributions of various immune cells, including Th cells, Treg cells, follicular helper T cells, and B cells, to the pathology of IgAN [109–114]. Within IgAN, these lymphocytes exert abnormal functions, which are significantly involved in the pathogenesis of IgAN, aggravating kidney inflammation and injury [111, 114, 115].

Dysfunctional newly synthesized lymphatic vessels contribute to the maleficent role of lymphangiogenesis in IgAN, similar to hyperglycemia-induced kidney disease. In addition, macrophages contribute to abnormal lymphangiogenesis in IgA glomerulonephritis. Emerging evidence demonstrates that CD137 ligand (CD137L)-secreted macrophages are present in IgA nephropathy, similar to other chronic kidney inflammatory diseases. CD137L interacts with CD137 on lymphatic endothelial cells, inducing lymphatic autophagy and lymphangiogenesis [116]. It may significantly contribute to the dysfunctional kidney lymphatic vessels, resulting in loss of transportation of inflammatory-associated molecules and obstruction of lymphatic routes for immune cell migration. Eventually, this CD137L-CD137 pathway drives fibrogenic responses, resulting in kidney fibrosis.

#### Lupus nephritis

Lupus nephritis is significantly associated with the progression of kidney inflammation and fibrosis. Still, only a few studies have focused on lymphangiogenesis's specific role in lupus nephritis. Inhibition of lymphangiogenesis in a mouse model of lupus nephritis (LN) distinctly alleviated the severity of the disease, but the effect of lymphangiogenesis was confounded in this model [117]. Additionally, a recent study found that kidney lymphangiogenesis induced the trafficking of LN-specific Mono/MΦ to both the entry and exit of the injured lesion [118]. The maleficent effect of lymphangiogenesis is probably related to the positive feedback between the renal inflammation site and the dLN, as mentioned above. However, whether the protective role of lymphangiogenesis can alleviate lupus nephritis remains unclear. In line with other chronic kidney diseases, lymphangiogenesis may mediate kidney injury through the clearance of immune and inflammation-related molecules and mediation in immune cell trafficking.

#### Hypertensive nephropathy

Patients and animal models with hypertension manifest a substantial increase of activated immune cells in the kidney [14, 119–126]. Infiltration of activated macrophages, DCs, B cells, and T cells distinctly aggravates renal injury and fibrosis, exacerbating sodium retention and ulteriorly elevating blood pressure [64, 127]. In general, the inflammatory response in the kidney, which is triggered by hypertension, further deteriorates both kidney function and hypertension condition [120].

In the setting of HTN, inflammation-associated kidney lymphangiogenesis is significantly induced [14, 119, 120, 128–130]. Previous studies demonstrated that HTN stimuli indirectly promote lymphangiogenesis instead of prompting LECs proliferation. HTN stimuli interact with various immune cell-secreted factors, sprouting lymphatic vessels [131]. Notably, increased extracellular ions in the kidney may directly activate macrophages to facilitate kidney lymphangiogenesis in hypertensive conditions. The mechanism above was discovered earlier in the dermis interstitium. Studies have demonstrated that increased osmolarity and extracellular salts within the skin directly activate TonEBP in macrophages and DCs, further inducing macrophages to express VEGF-C, which promotes lymphangiogenesis.

Given the critical role of lymphangiogenesis in fluid clearance and the significantly increased kidney lymphangiogenesis in HTN models, the interaction between the lymphatic system and HTN has drawn much attention. Furthermore, despite newly synthesized lymphatics' function in modulating renal fluid homeostasis, recent evidence supports that lymphangiogenesis can also influence HTN by regulating the immune response in the kidney. Enhancement of kidney lymphangiogenesis exerts a protective effect against hypertension, reducing renal immune cell accumulation and alleviating inflammation [14, 45, 120, 128, 130, 132, 133]. Despite clearance of excessive fluid, lymphangiogenesis also elevates drainage of infiltrated immune cells and pro-inflammatory cytokines secreted by these cells. In several hypertension mouse models with kidney-specific overexpression of VEGF-D (KiD^−^VD^+^ mouse model), excessively accumulated immune cells, including macrophages in the kidney, were all reduced, subsequently preventing hypertension [132]. Furthermore, in hypertension conditions, kidney lymphangiogenesis also actively regulates the migration of immune cells, including macrophages, DCs, and T cells, via increased secretion of CCL21 and CCR7. An angiotensin II-induced hypertension (A2HTN) mouse model study has proved that lymphangiogenesis significantly reduces the CD11c^+^F4/80^−^ monocyte renal population [130]. Activated monocytes in the kidney express pro-inflammatory cytokines and mediate T-cell activation and differentiation. Naïve T cells differentiate into Th1 or Th17 cells, which secrete pro-inflammatory and pro-hypertensive cytokines, causing sodium retention and hypertension [134]. Thus, reducing these monocyte populations in kidney attributes inhibits excessive inflammation response in the kidney due to hypertension.

However, evidence supports that renal-specific lymphangiogenesis cannot fully rescue kidney hypertensive condition but can only alleviate systemic blood pressure [135]. Additionally, as Treg cells can inhibit inflammation response and improve sodium retention within HTN [75, 136], in line with other kidney diseases, lymphangiogenesis-induced removal of Treg cells might aggravate kidney inflammation and hypertension conditions.

In the HTN setting, kidney lymphangiogenesis is limited in renal immune cell trafficking [132]. Additionally, due to current evidence, an uneven outcome in the transport level of different immune cell populations by newly formed renal lymphatic vessels has been discovered in hypertension models. The great involvement of T cells and M1 macrophages in hypertensive kidneys possibly results in limited transferring of these cells from kidney tissues through kidney lymphangiogenesis, compared with other renal immune cell populations.

**Fig. 2:**
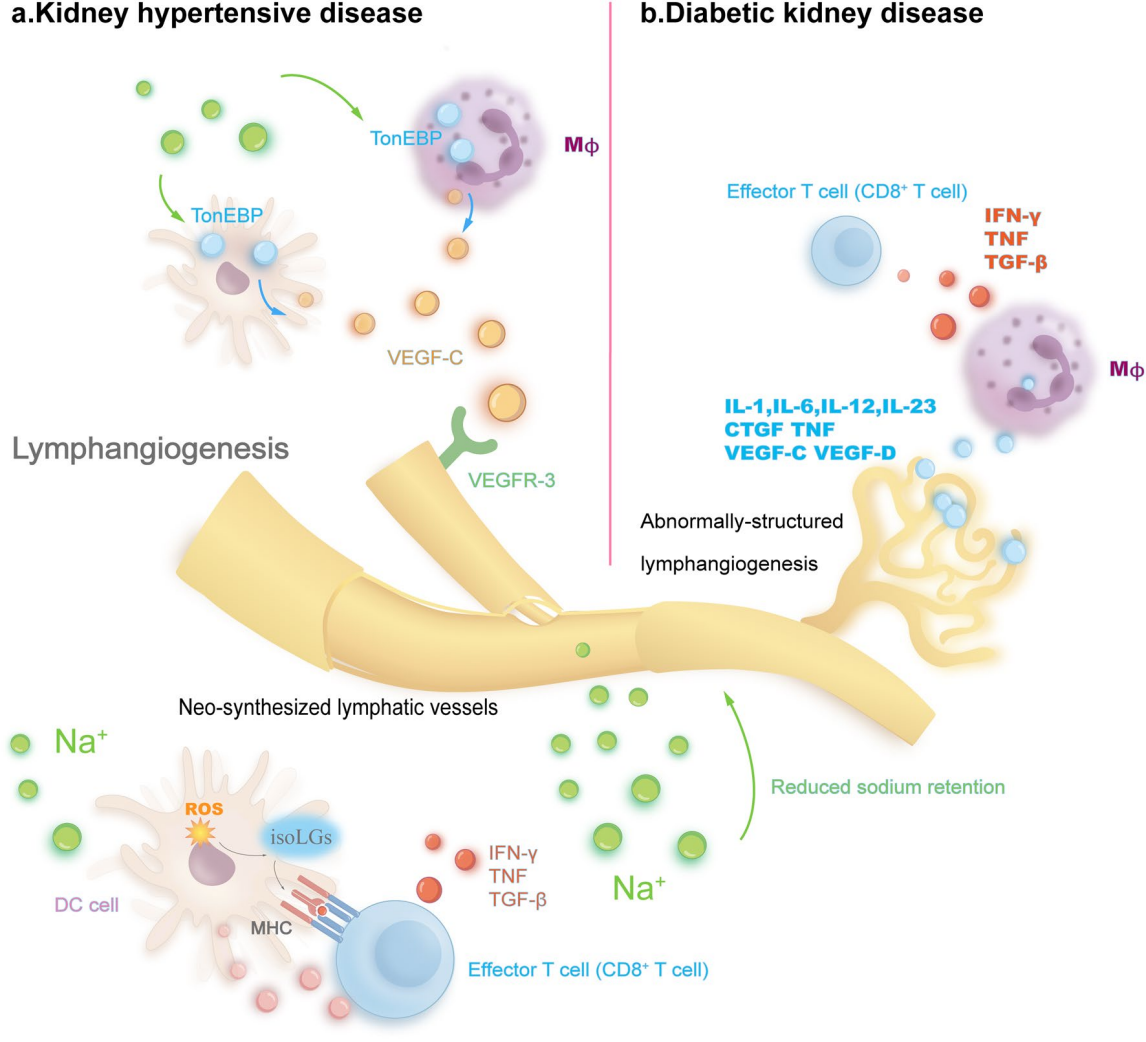
Distinct mechanisms of lymphangiogenesis in certain kidney disease. **a**. In kidney hypertensive disease, sodium retention induces lymphangiogenesis through a Na +—TonEBP—VEGF-C pathway. Na + directly activates transcription factor of tonicity responsive enhancer-binding protein (TonEBP) in macrophages and dendritic cells (DCs) to promote expression of VEGF-C from macrophages, and then induces lymphangiogenesis. Sodium retention can directly activate DCs to express cytokines for further antigen-specific T cell accumulation and activation. Na + enters dendritic cells, subsequently leading to Ca2 + influx and then activation of protein kinase C, eventually resulting in increased expression of reactive oxygen species (ROS). ROS oxidates fatty acids into isolevuglandins (IsoLGs), which activates dendritic cells to produce proinflammatory cytokines (IL-1β, IL-6, IL-23) and activate T cells to proliferate and express inflammatory cytokines including TNF, IFN-γ and TGF-β. Lymphangiogenesis can reduce sodium retention, therefore inhibits DCs activation and the inflammatory response. **b**. In diabetic kidney disease, excessive cytokines expressed during the chronic inflammation condition create a specific microenvironment, which significantly induces abnormally-structured lymphangiogenesis

Interestingly, unlike other kidney diseases, in hypertension condition, kidney lymphangiogenesis can also directly regulate immune cell activation through sodium transport (Fig. 2). Recent study has revealed that kidney lymphangiogenesis directly suppresses activation and accumulation of DCs through reducing Na^+^ retention, consequently relieving hypertension and mitigating the progression of HTN [132]. As Na^+^ stimulation can activate DCs [137], reduction of Na^+^ retention through enhanced kidney lymphangiogenesis can directly inhibit DCs activation.

#### Polycystic kidney disease

Unlike the early stage of kidney inflammation, M2 macrophages are predominant macrophages within PKD, which can promote tissue repair and are ultimately profibrotic [138]. Of note, M2 macrophages distinctly enhance cyst enlargement in PKD [139]. Studies have demonstrated that kidney lymphangiogenesis can remodel vessel structure, expand lymphatics to transport accumulated fluid in cysts and inhibit cyst progression [140]. However, few study concentrates on the transportation of local immune cells or the immune regulatory function of lymphangiogenesis within PKD. In line with other kidney diseases, lymphangiogenesis may alleviate the progression of PKD by transporting inflammatory cells and reducing M2 macrophage infiltration surrounding the cysts. However, available evidence has indicated a protective role of CD8^+^ T cells in the context of PKD [141]. Further studies are required to investigate the immune regulatory role of kidney lymphangiogenesis and its overall impact on PKD.

#### Kidney transplantation

After kidney transplantation, the mutual interaction between the allograft and the recipient’s immune system may generate a series of immune responses, resulting in transplant rejection. Immune cells are trafficked between the kidney allograft and the recipient’s original system via blood circulation and newly formed lymphatic vessels [142, 143]. Studies with conflicting results have revealed that lymphangiogenesis can both serve a protective role and a maleficent role in transplant rejection. Kidney allografts with greater density of kidney lymphatic vessels are less likely to generate renal interstitial fibrosis and renal tubule atrophy [144]. Likewise, promoting lymphangiogenesis in the kidney allograft significantly alleviates transplant rejection and extends the mice recipient's survival time [145]. Vigorous neo-synthesized lymphatic vessels transport inflammatory cells, clearing interstitial edema and subsequently protecting the kidney allograft [145–147]. In line with acute kidney injury, lymphatic regulation of immune cells via lymphangiogenesis protects recipients from transplant rejection. Kidney lymphangiogenesis can directly suppress local CD8^+^ T cell's immune response in kidney allograft via PD-L1, which is secreted by LECs [148]. In contrast to AKI, lymphatic migration of Treg cells may suppress allograft rejection [149].

Inversely, several studies have suggested that the expansion extent of lymphatic vessels positively correlates with the severity of transplant rejection [144, 150, 151]. In addition, it is demonstrated that inhibiting lymphangiogenesis can mitigate the injury of allografts [152], while ligation of lymphatic vessels benefits transplant rejection. Neo-synthesized lymphatic vessels have an abundant accumulation of CD 45^+^ lymphocytes, mainly MHCII^+^, ED-1^−^, IDO^−^, HIS13^−^, and CD103 antigen-present cells. The increase of these antigen-presenting cells can exacerbate injury of transplant rejection in kidney allografts [153]. Increasing recruitment of antigen-presenting cells in recipient dLNs initiates the alloimmune response, leading to inflammatory cell infiltration in the kidney allograft and further destruction of the structure and function of the allograft [154].

## Conclusion

Despite an increased emphasis on the immunological regulatory function of lymphangiogenesis in renal disorders, there are still notable gaps in our understanding. A substantial body of evidence indicates that kidney lymphangiogenesis plays a significant part in immunological regulation, with its effects being either beneficial or detrimental depending on the specific kidney condition under consideration. Recent studies have elucidated several immunological functions of lymphatic veins for specific kidney disorders. Nevertheless, existing research predominantly examines renal inflammation, fibrosis, and a restricted range of prevalent kidney disorders, such as HTN and DKD. Furthermore, these investigations primarily rely on animal models as the primary means of study. Moreover, a more comprehensive examination is required to elucidate the underlying factors contributing to the varied impacts of lymphangiogenesis on distinct renal disorders. There is an urgent need for a more extensive comprehension of the immunological regulatory processes underlying renal lymphangiogenesis within the framework of kidney disease. Examining these complex pathways can yield new perspectives in developing therapeutic interventions that specifically target the beneficial aspects of lymphangiogenesis or reduce its detrimental impact on renal disorders.

## Data Availability

Not applicable.

## Abbreviations

LEC: Lymphatic endothelial cell
AKI: Acute kidney injury
CKD: Chronic kidney disease
DC: Dendritic cell
dLNs: Draining lymph nodes
DKD: Diabetic kidney disease
ESKD: End-stage kidney disease
HUS: Haemolytic uraemic syndrome
VEGF-C: Vascular endothelial growth factor C
VEGF-D: Vascular endothelial growth factor D
VEGF-3: Vascular endothelial growth factor receptor 3
VEGFR3: Vascular endothelial growth factor receptor 3
NRP2: Neuropilin 2
TGF-β: Transforming growth factor β
CTGF: Connective tissue growth factor
LYVE-1: Lymphatic vessel endothelial receptor-1
A2HTN: Angiotensin II-induced HTN
CCR7: Chemokine (C–C motif) receptor 7
CCL19: Chemokine (C–C motif) ligand 19
CXCL: Chemokine (C-X-C motif) ligand
CCL: Chemokine (C–C motif) ligand
ICAM1-1: Intercellular adhesion molecule 1
VCAM-1: Vascular cell adhension molecule
VE-Cadherin: Vascular endothelial cadherin
IL-1β: Interleukin-1β
TNF-α: Tumor necrosis factor-α
IL-10: Interleukin-10
TonEBP: Transcription factor tonicity-responsive enhancer-binding protein
HEV: High endothelial venule
HTN: Hypertensive nephropathy
IgAN: IgA nephropathy
IFN-γ: Interferon γ
LHTN: L-NAME-induced HTN
PKD: Polycystic kidney disease
T_RCM_ cells: Recirculating memory T cells
Treg cell: Regulatory T cell
SSHTN: Salt-sensitive HTN
S1P: Sphingosine-1-phosphate
TCR: T cell antigen receptor
Th cell: T helper cell
Th1 cell: T helper cell 1
Th17 cell: T helper cell 17
PD-L1: Programmed death-ligand 1
UUO: Unilateral ureteral obstruction
ROS: Reactive oxygen species
DAMPs: Danger-associated molecule patterns
CD137L: CD137 ligand
